# The impact of food aid interventions on food insecurity, diet quality and mental health in households with children in high-income countries: a systematic review

**DOI:** 10.1017/S1368980024001769

**Published:** 2024-10-04

**Authors:** Charlotte Stahacz, Nisreen A Alwan, Elizabeth Taylor, Dianna Smith, Nida Ziauddeen

**Affiliations:** 1 School of Primary Care, Population Sciences and Medical Education, Faculty of Medicine, University of Southampton, Southampton, UK; 2 NIHR Applied Research Collaboration Wessex, Southampton, UK; 3 NIHR Southampton Biomedical Research Centre, University of Southampton and University Hospital Southampton NHS Foundation Trust, Southampton, UK; 4 School of Geography and Environmental Science, University of Southampton, Southampton, UK

**Keywords:** Food bank, Food pantry, Food insecurity, Diet quality, Households with children

## Abstract

**Objective:**

Households with children accessing food aid in high-income countries are often food insecure. We aimed to review the evidence on food aid interventions in households with children and impact on food insecurity, diet quality and mental health.

**Design:**

A systematic search was conducted using Web of Science, MEDLINE, CINAHL and PsycINFO. Articles published from January 2008 to July 2022 including cross-sectional, cohort and interventional studies in high-income countries were eligible.

**Setting:**

Food aid is defined as the use of interventions providing free food items by community and/or charitable organisations.

**Participants:**

Two-parent, lone parent or households with a primary caregiver with at least one child ≤ 18 years.

**Results:**

From a total of 10 394 articles, nine were included. Food banks, mobile pantry combined with a free meal for children, backpack provision during school term and food parcel home delivery interventions were evaluated. Food bank models offering additional support such as community programmes, health and social services, cooking classes and free meals for children, client-choice-based models and programmes providing convenient access were associated with improved food security and diet quality (increased intake of wholegrains, fruit and vegetables). One study reported an improvement in mental health and food bank access at the end of 18 months but not at earlier timepoints and one study reported no change in parents’ mental health.

**Conclusions:**

Accessing food aid was linked to improved diet quality and reduced food insecurity in some studies. Allowing clients to choose food items and providing support services were most effective.

Food security refers to whether households can consistently afford and have physical and economic access to sufficiently healthy food at all times^(1)^. Approximately 12 % of households in the UK reported being food insecure between 2021 and 22^(2)^. In the United States of America (USA), 10·2 % of households and 12·5 % of households with children were food insecure in 2021^(3)^. Figures from Canada were slightly higher at 18·4 % in 2021^(4)^. Data from public surveys in the UK showed that food insecurity in households with children increased from 12·1 % in January 2022 to 23·4 % in June 2023^(5)^.

Food aid, where food is free or greatly reduced in price, in high-income countries is usually provided by charitable organisations. The continuing financial crisis and global food inflation are leading to rising demand for food aid^(6)^. In the USA, 49 million people required food aid in 2022^(7)^. In the UK, people using food banks increased by 177 % from March 2019 to 2020^(8)^. More recently, almost 3 million food parcels were distributed by the largest group of food banks between 1st April 2022 and 31st March 2023, an increase of 37 % from the same period in the previous year^(9)^. The trend is reflected in Canada, with almost 1·5 million visits to food banks between March 2021 and March 2022, an increase of 15 % from the previous year^(10)^. The pressures of the economy are also affecting food aid, with food banks facing challenges of declining donations, increasing numbers of people requiring support and sustaining their volunteer workforce^(11)^. Research has identified barriers and limitations of food banks, such as limited opening hours, inadequate food provisions^(12)^ and feelings of shame and embarrassment among users^(13,14)^. Interventions providing emergency access to food are subsequently evolving to try and better serve users’ needs.

The need for food aid could be a consequence of inadequate welfare assistance resulting in insufficient resources to purchase food or short-term ‘shocks’ such as loss of income due to job loss, illness or disability. Low-income households are particularly vulnerable to food insecurity^(15–18)^. Evidence shows people experiencing food insecurity are more likely to experience unemployment, low income, be of non-white ethnicities, have low educational qualifications, be lone-parent households and have a disability^(18–21)^. Food bank use, food insecurity, poverty and adverse health outcomes are closely related^(22)^. Food insecurity is associated with an increased risk of chronic diseases such as CVD^(23)^, type 2 diabetes and poor mental health^(24,25)^.

Household food insecurity is complex as one or all family members can experience food insecurity at different severities with a range of implications. Adults in food-insecure households have been observed to skip or reduce their meals to ‘shield’ children from the effects of hunger and undernourishment leading to a detrimental effect on the adult’s diet quality^(26)^. Children living in food-insecure households have a poor-quality diet^(27)^ with low consumption of fruits and vegetables^(28)^. Low fruit and vegetable consumption are risk factors for CVD, cancer and all-cause mortality^(29)^. Children in food-insecure households also have a greater risk of mental health problems^(30,31)^, but shielding has been observed to improve mental health outcomes in children^(32)^. Associations have been found between food insecurity and behavioural problems, poor academic performance and emotional problems^(33)^. Subsequently, food insecurity and associated poor-quality diet and mental health problems can place a major financial strain on the healthcare system in treating short-term and chronic conditions leading to a public health crisis.

Few studies have examined the effectiveness of multiple types of food aid interventions, and existing studies predominantly focus on outcomes in adults receiving food aid^(12,34,35)^. To address this gap, we broadened the interventions to cover various types of food aid and included outcomes in children. Therefore, this review aims to systematically review and narratively synthesise studies investigating the impact of food aid interventions in households with children (≤ 18 years) in high-income countries. The first objective is to investigate the effectiveness of food aid interventions in reducing food insecurity. The second objective is to investigate how food aid interventions impact diet quality, mental health and/or weight status in adults and children within a household.

## Methods

This systematic review is reported according to the Preferred Reporting Items for Systematic Reviews and Meta-Analysis guidelines^(36)^. A scoping search was initially conducted in the Web of Science to identify keywords commonly describing food aid interventions. Synonyms for the category’s population, food aid, food insecurity, diet quality, mental health and weight status were identified. Synonyms were combined with ‘OR’ and categories with ‘AND’ shown in Table 1, creating a comprehensive search. A library specialist assisted with developing the search strategy. A systematic electronic search was conducted on 09th July 2022 using the databases Web of Science and EBSCOhost for MEDLINE, CINAHL and PsycINFO.

**Table 1:** Search strategy

Category for search term	Search terms
Population (All terms combined with ‘OR’)	Child*, infant, toddler, baby, babies, school age*, newborn, pre school, preschool, famil*, lone parent, single parent, household* young child* primary caregiver, parent*, teen*, adolescent, young adult, young person, young people
Food aid interventions (All terms combined with ‘OR’)	Food bank*, foodbank*, food pant* food aid, food assistance, food shel*, community food program*, emergency food, food parcel, community shop, charit* food assistance, food supply, food stor*
Diet quality (All terms combined with ‘OR’)	Food intake, food quality, diet*, diet* quality, diet* adequacy, diet* intake, nutrition*, nutrition* intake, nutrition* adequacy, nutrition* wellbeing, nutrition* survey, nutrition* quality, nutri*requirements, nutrition* status, nutri* value, energy intake, macronutrient, micronutrient, vegetable, fruit, diet* fat, fibre, fiber, vitamin, mineral, dairy, child* nutrition*, infant food, infant nutrition*, calor* intake
Food security (All terms combined with ‘OR’)	Food insecurity, food security, hunger, food insufficiency, poverty, nutrition* security, food poverty, food deprivation, food sufficiency
Mental health (All terms combined with ‘OR’)	Mental health, anxiety, depression, stress
Weight status (All terms combined with ‘OR’)	Weight, Underweight, overweight, obes*, BMI, body mass index

### Eligibility criteria

The search was limited to studies published in English from 1st January 2008 to 9th July 2022 to ensure up-to-date interventions are included. The global financial crisis of 2007–2008 resulted in widespread job losses, a substantial rise in food insecurity and an increased demand for food aid in high-income countries^(37,38)^. Food aid has since remained a key resource for people living in poverty or facing a short-term financial crisis^(39,40)^. Detailed inclusion and exclusion criteria are provided in Table 2.

**Table 2:** Inclusion and exclusion criteria for the review

Inclusion criteria	Exclusion criteria
Case studies, cross-sectional, longitudinal cohort, randomised controlled trials and mixed method studies	Systematic or other review articles, dissertations, conference abstracts and qualitative studies.
Households (two-parent, lone parent or any primary caregiver) with at least one child from 0–18 years of age	Populations with chronic disease, the elderly, students, homeless or adult households with no children over 18 years of age
Populations in high-income countries as defined by the World Bank^(87)^	Low-and medium-income countries
Food aid considered as the use of food banks or other interventions providing free food items by the community and/or charitable organisations	State welfare food assistance programmes, pre-prepared meals such as soup kitchens, community-supported agriculture programmes/gardens and subsidised and/or reduced price food items
Quantitative measures of diet quality (dietary intake such as food groups, comparison to nutritional guidelines, dietary reference values or against scoring systems); food security and mental health (scores or using surveys and questionnaires)	Qualitative studies

### Screening process

Results were exported into Rayyan^(41)^, an online software screening tool, and duplicates were removed. Title and abstract screening was performed by a single reviewer (CS); however, a random 10 % sample was independently screened by a second reviewer (ET). An agreement of 94·1 % was achieved between the two reviewers, and discrepant titles were included in the abstract screening. CS reviewed the remaining 90 % of titles. The same process was followed for abstract screening with 91 % agreement, and discrepant abstracts were included for full-text screening. CS and ET independently screened all remaining full-text papers against the inclusion criteria. The agreement was 64 %. CS and ET discussed the eight studies which were discrepant and reached a consensus for 4 papers. A third reviewer (NZ) was consulted regarding eligibility for the remaining 4 discrepant papers.

### Data extraction

CS extracted the data from the full-text papers; however, a random selection of 20 % from the final full-text papers was selected for second reviewer extraction. CS and ET independently extracted data for these papers using a modified version of the Cochrane Collaboration data extraction form^(42)^. CS and ET reviewed the information to ensure consistency. Data extracted included authors, year, country, study design, population, sample size, description of intervention, data collection method and outcomes. For statistically significant outcomes, CI or *p*-values were reported.

### Quality assessment and risk of bias assessment

CS and ET independently conducted quality assessment and risk of bias for all full-text papers using the National Heart Lung and Blood Institute Assessment tool^(43)^. Studies were categorised as good, fair or poor. A ‘good’ study would have a low risk of bias.

## Results

**Fig. 1:**
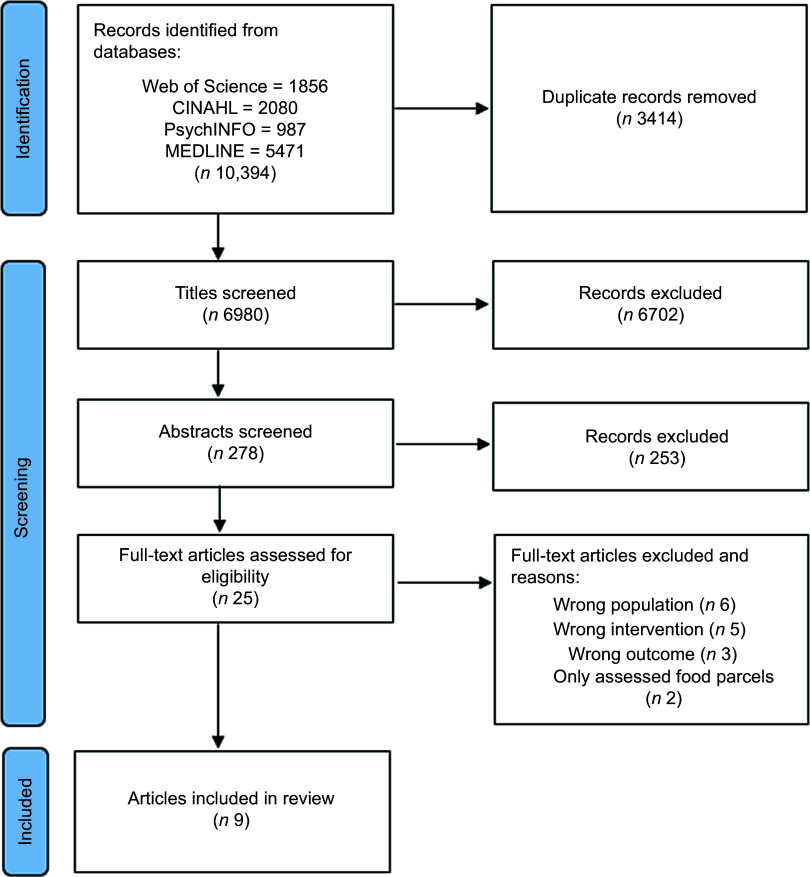
PRISMA flow chart detailing the selection process

The search identified 10 394 records, of which 3414 were duplicates. Titles of 6980 records were screened, and of these, 278 abstracts were screened. Full-texts of 25 papers were screened and 9 papers were included in this review (Fig. 1). Due to the heterogeneity of the studies, a meta-analysis could not be performed and the results are presented as a narrative review.

### Characteristics of included studies

Study designs include one cluster randomised controlled trial (RCT), which reported relevant outcomes in two separate papers^(44,45)^, three cross-sectional^(46–48)^ and four cohort^(49–52)^ studies (Table 3). Two studies were based in Canada^(49,50)^ and seven in the USA^(44–48,51,52)^.

**Table 3:** Characteristics of included studies

Study characteristics							Measures and outcomes				
First author, year	Study design	Country & Sample size	Population				Inclusion criteria	Intervention/Exposure	Food Insecurity	Diet quality	Mental Health
			Age	Gender	Ethnicity	Households with children					
Briefel, 2021^(45)^	Cluster randomised control trial^*^	Oklahoma, USA 40 school districts (20 treatment, 20 control) 2859 households (1340 treatment, 1519 control)	< 40 64 % > 40 36 %	Not reported	Hispanic 12 % White 57 % Black 18 % Native American 14 %	All households Average number of children in households 2·5	Households with children aged ≥ 4 eligible for free school meals Or from schools where all children receive free meals in a participating school district	Choice of 5 food parcels available to order and delivered monthly for 25 months Each parcel included a $15 cheque for fresh/frozen fruit and vegetables (FV)	18-item US Household Food Security Module Children, adult and household food insecurity	Reported in Cabili, 2021^(44)^	–
Cabili, 2021^(44)^	Cluster randomised control trial^*^	Oklahoma, USA 40 school districts (20 treatment, 20 control) 2859 households (1340 treatment, 1519 control)	< 40 64 % > 40 36 %	Not reported	Hispanic 12 % White 57 % Black 18 % Native American 14 %	All households Average number of children in households 2·5	Households with children aged ≥ 4 eligible for free school meals Or where all children receive free meals in a participating school district	Choice of 5 food parcels available to order and delivered monthly for 25 months Each parcel included a $15 cheque for fresh/frozen FV	Reported in Briefel, 2021^(45)^	Children’s diet quality NCI 26-item screener FV, FV without fried potatoes, fruits, vegetables, vegetables without fried potatoes, wholegrains, added sugars from foods and beverages, added sugars from sugar-sweetened beverages (SSB) Intake compared to USDA 2015–2020 Dietary Guidelines for Americans	–
Chiappone, 2021^(46)^	Cross-sectional	Nebraska, USA *n* 563 households	19–28 26 % 29–34 24 % 35–43 25 % 44–67 25 %	Female 78 %	Non-Hispanic Black 40 % Non-Hispanic White 25 % Hispanic 19 %	All households Number of children in household 0–2 63 % > 2 37 %	Low-income families ≥19 years of age and a parent or primary caregiver to a child at least 50 % of the time	Frequency of food pantry use: • Frequent user (once a month, once a week, multiple times a week) • Semi-frequent user (once or twice a year) • Non-user (Do not utilise food from a pantry)	USDA 6-item short-form Food Security Module	NCI 24-item Family Life, Activity, Sun, Health and Eating screener (FLASHE) screener Food groups: FV with potatoes, FV without potatoes, foods with added sugar, SSB Intake adapted to frequency of intake: < 1 times per day ≥ 1 times per day	–
Fan, 2021^(47)^	Cross-sectional	USA *n* 134 (67 charitable food assistance clients, matched to 67 non-clients)	Mean age (sd): 50 (±14·7)	Female 81 %	White 72 % Black 19 % Hispanic 11 %	% of households with children not reported Mean number of children < 18 years (sd) = 1·3 (±2·0)	Data from the 2021 USDA National Household Food Acquisition and Purchase Survey (FoodAPS)	Households that utilised charitable food assistance within a randomly selected 7-day survey week (April 2012–January 2013)	USDA’s 30-day Adult Food Security Scale	Ratio of pounds of food consumed against pounds of food obtained by each household compared to recommendations from the USDA Thrifty Food Plan (TFP)	–
Loopstra, 2012^(49)^	Cohort	Toronto, Canada *n* 371	Not reported	Not reported	Not reported	All households Number of children in household: 1 35 % 2 32 % 3 21 % 4 + 9 %	Low-income families, at least 1 child ≤18 years of age, living in a rented dwelling for at least one month Gross income at/below mid-level of Statistics Canada’s 5-category income adequacy scale^†^	Food bank use and frequency	18-item Household Food Security Survey Module (HFSSM)	–	–
Palakshappa, 2021^(52)^	Cohort	North Carolina, USA *n* 41 (parent-child dyads)	Mean age (range) = 46 years (19–78)	Female 49 % Child gender: Male 46 % Female 54 %	Non-Hispanic White 2 % Non-Hispanic Black 91 % Hispanic 7 %	All households Mean child age (range): 10 years (4–18) Mean number of children in the home (sd) 2·0 (±1·3)	Residents of the Boston-Thurmond neighbourhood ≥18 years of age or older At least one school-aged child (4–18 years of age)	Mobile pantry with food parcels (2–3 d of food for the family) available for weekend collection, optional cooking classes Mobile food truck providing children a free lunch (sandwich, fruit, grain and healthy dessert)	2-item Hunger Vital Sign Semi-structured interviews	NCI All-day FV Screener Intake compared against average daily MyPyramid servings	Patient-Reported Outcomes Measurement Information System (PROMIS) 8-item Short-Form
Rizvi, 2021^(50)^	Cohort	Ottawa, Canada Baseline *n* 401 Final follow-up *n* 271	Mean age 44 years^§^	Men 41 % Women 51 % Gender diverse 9 %	White 53 % First Nations/Metis/Inuit 9 % Visible minority 38 %	Dependent:^‡^ None 52 % One or more 41 %	People accessing community food banks in Ottawa ≥18 years of age	Different food bank models; Integrated within a Community Resource Centre, Choice distribution model and those with additional onsite programmes Baseline and 3 follow-up surveys at 6-month intervals – total duration of 18 months	HFSSM 18-item survey	–	12-item Short-Form Health Survey (Version 2)
Wright, 2018^(51)^	Cohort	Florida, USA Parents: *n* 52 Children: *n* 120	Not reported	Not reported	African American 76 %^§^ Hispanic 17 % Caucasian 5 %	All households	All students and their parents at the selected school were considered eligible	Backpack containing breakfast cereal, shelf-stable milk, a protein such as canned tuna, juice boxes, pasta, fruit cup, vegetables granola bars and fresh produce provided to all students in the school at the end of the day each Friday	Parental evaluation included the USDA Food Security Survey Children’s survey included questions such as, ‘Do you come to school without eating breakfast because there wasn’t enough food at home?’ ‘Do you worry about not having enough food to eat?’	–	Parental surveys included questions such as ‘Do you feel your child worries more than usual?’ ‘Do you feel your child is sad or depressed?’ Children’s survey included, ‘Do you get angry a lot at school?’ ‘Are you sad a lot?’
Zigmont, 2022^(48)^	Cross-sectional	Connecticut, USA *n* 153	Not reported	Female 86 % Male 14 %	Not reported	Households with children < 18 years of age 78 % Number of children in household: 0 22 % 1 12 % 2 28 % 3 21 % ≥4 17 %	Any persons accessing the Mobile Food Pantry at the 4 locations	Mobile food pantry truck at selected sites providing fresh produce, bread and non-perishables A second mobile truck visits the same site providing dinner (sandwich, drink and fruit)	The following question was asked: ‘In the past month, was there any day when you or anyone in your family went hungry because you did not have enough money for food?’	Measured by the question ‘Summer meals helps your family eat a healthier diet’	–

Food parcels could be ordered online or via telephone. Choice of 5 food parcels containing shelf-stable foods, including 6 protein-rich items, 2 dairy items, 4 grain foods, 4 cans of fruit, 12 cans of vegetables, recipes and nutrition education handouts. All eligible children were allowed 1 parcel each. Chickasaw Nation Nutrition Service nutritionists selected items based on the quality of their nutritional content, knowledge about what Chickasaw Nation families eat and communication with Chickasaw families. The food parcel, including the $15 check, was valued at $53 per eligible child^(44,45)^.

* The RCT studies are the same intervention with food security reported by Briefel *et al.* ^(45)^ and children’s diet quality by Cabili *et al.* ^(44)^

† Statistics Canada’s 5-category income adequacy scale: ≤ $29 999, $39 999 or $59 999 if household 1 or 2 people, 3 or 4 people or 5+ people, respectively.

‡ Dependents include children or adult dependents.

§ Ethnicity data shown are for the whole school population (*n* 496) and not the sample population. Socio-demographic sample data was not collected as the researchers were concerned about the privacy and confidentiality of the participants. The data indicate the ethnicity mix of the school.

The study population varied widely within the studies. Generally, females were the main respondents^(46–48,50)^. Three studies were in ethnic minority groups, predominantly non-Hispanic Black populations^(46,51,52)^. Four were in mostly non-Hispanic White populations^(44,45,47,50)^. Two studies^(48,49)^ did not collect individual ethnicity data; however, one stated the residents in the target neighbourhoods were mostly Black or Hispanic^(48)^. The populations were mostly from low-income areas^(44–46,48,49,51,52)^ and/or from neighbourhoods where most children were eligible for free school meals^(48,51)^.

Two studies evaluated a mobile pantry combined with providing a free meal for children; one operated on weekends^(52)^ and the other during summer holidays^(48)^. One study evaluated a programme where children were provided free food provisions in a backpack during the school term^(51)^. Four studies assessed food bank models/use^(46,47,49,50)^. The RCT analysed a free food parcel home delivery intervention^(44,45)^.

The parcel delivery^(44,45)^ and backpack^(51)^ interventions were primarily aimed at children. Two programmes^(48,52)^ aimed to benefit the whole household by locating a mobile pantry and food truck giving free meals to children in the same location. Four studies investigated food aid use, two included households comprising any mix of individuals^(46,49)^, and two only investigated participants with children^(47,50)^.

Food insecurity was reported as a quantitative outcome in seven studies^(45–51)^. One study^(52)^ collected qualitative data from 20 participants using semi-structured interviews to investigate the impact of food insecurity on families and perceptions of the effectiveness of the programme. Data were collected using validated questionnaires; two studies used the United States Department of Agriculture (USDA) 18-item Household Food Security Module^(45,51)^, one used the 6-item Short-Form Household Food Security Module^(46)^ and one utilised the USDA 30-day Food Security Scale^(46)^. Zigmont et al.^(48)^ used one specific question from the USDA 18-item Household Food Security Module to assess food insecurity. The two studies from Canada utilised the 18-item Household Food Security Survey Module^(49,50)^, an adapted version of the USDA 18-item Household Food Security Module which has been used routinely by the Canadian government^(53)^.

Dietary data were collected in five studies^(44,46–48,52)^. Two different screeners from the National Cancer Institute were used in two separate studies: 24-item fruit and vegetable screener^(46)^ and an all-day fruit and vegetable screener^(52)^. Another study collected food group data consisting of fruit and vegetables, foods with added sugar and sugar-sweetened beverages and compared intake to USDA dietary guidelines^(44)^. One cross-sectional study^(47)^ used data from the 2012 National Food Acquisition and Purchase Survey (FoodAPS). The researchers compared the ratio of the USDA Thrifty Food Plan recommended pounds of consumption and actual pounds of foods obtained by the household for each food group. The Thrifty Food Plan is designed by the USDA to meet the nutritional requirements of a family of four, integrating USDA healthy eating guidelines and food preferences and is achieved at the lowest cost^(54)^. One study^(48)^ collected limited dietary data, asking respondents whether they strongly agree, agree, disagree or strongly disagree if ‘Summer meals help my family eat a healthier diet.’ The figures were presented alongside other socio-demographic characteristics of the sample by food security status.

Mental health outcomes were measured in three studies^(50–52)^. Two studies used validated questionnaires: Patient-Reported Outcomes Measurement Information System 8-item Anxiety Short-Form to assess parental anxiety^(52)^ and the 12-item Short-Form Health Survey, version 2 to evaluate the mental health of the adult respondents^(50)^. One study assessed mental health and anxiety by providing a short questionnaire to both children and parents asking about the children’s mental health and anxiety^(51)^.

### Quality assessment

There was high heterogeneity between the study populations, with various measures and reporting of diet quality and mental health outcomes. The RCT^(44,45)^ was the only study rated ‘good’. Blinding was not possible as the intervention involved participants ordering a food parcel. Randomisation of households was carried out to reduce confounding factors. There was low attrition of participants, ensuring the statistical power of the results was reliable. Four studies^(46,47,50,52)^ were rated as ‘fair’. Of these, two studies^(46,52)^ reported dietary intakes using validated surveys. One^(52)^ collected baseline and follow-up data after three to six months, with the other study^(46)^ collecting data at one point in time only during visits to community centres where participants were recruited. One study analysed household food purchasing data from a nationally representative survey of US households^(47)^.

Food security data for all studies were collected using a validated survey. Data from all studies were self-report, thereby introducing recall^(55)^ and response bias^(56,57)^ which can lead to over- or underestimating the true effectiveness of the interventions.

There are some limitations of the dietary data collection for all the studies. Diet surveys were collected retrospectively, and therefore liable to information bias, and used either a 30-day^(44,52)^ or one-week^(46,47)^ reference period for analysis. Two studies^(44,52)^ investigating children’s diet quality asked the parent/caregiver to report their child’s food consumption. Another source of information bias is that parents/caregivers may not be present for all of their children’s eating occasions, leading to incomplete or inaccurate data.

In one study^(48)^, while dietary intake data were not collected, participants were asked whether the intervention helped their family eat a healthier diet. The question is too broad to elicit accurate data for determining diet quality, which limits the validity of these findings.

Dietary surveys were carried out using various methods across the studies, including interviews in-person^(48,52)^, via the phone^(44)^ or both^(47)^. These methods risk introducing social desirability bias, where participants may over-report the consumption of healthier foods, particularly with sensitive discussions regarding their children’s dietary habits.

Two studies^(49,51)^ did not provide socio-demographic data on the sample population. Low response rates ^(51)^, high attrition^(50,52)^ and lack of completion of follow-up surveys^(51,52)^ were key limitations. Due to high attrition in the Ottawa study^(50)^, the researchers reduced the study period from 24 to 18 months.

Convenience sampling was mostly used, which has a high risk of selection bias. Participants were recruited door-to-door^(49,52)^, whilst waiting in line at food banks^(48,50)^, from community venues^(46)^ and from parents expressing an interest in participating in the school backpack programme^(51)^.

### Summary of findings

The summary of findings for all included studies is presented in Table 4. In some studies, not all participants used food aid, and therefore, only the subsample that used food aid is included in the tables.

**Table 4:** Summary of findings for included studies

Author, Date	Food aid intervention	Food insecurity	Diet quality	Mental health
Briefel, 2021^(45)^	97 % of eligible households ordered a food parcel at least once during the intervention Average participation rate in monthly orders was 61 % 88 % of households redeemed at least 1 Fresh cheque 1st follow-up at 12 months, 2nd at 18 months	No reduction of child food insecurity at 1st or 2nd follow-up in treatment group Significant reduction by 2·8 % points in adult food insecurity (*P =* 0·002, 95 % CI: –4·8, –0·9) and 2·4 % points in household food insecurity (*P =* 0·003, 95 % CI: –4·1, –0·6) at first follow-up No significant difference at 2nd follow-up for adult or household food insecurity	Reported in Cabili, 2021^(44)^	–
Cabili, 2021^(44)^	Reported in Briefel, 2021^(45)^	Reported in Briefel, 2021^(45)^	Children in the treatment group increased daily: Total fruit and vegetable consumption of 0·1-cup equivalents (95 % CI: 0·06, 0·13), 0·05-cup equivalents of fruits (95 % CI: 0·03, 0·08), 0·03-cup equivalents of vegetables (95 % CI: 0·01, 0·05), 0·07-ounce equivalents of wholegrains (95 % CI: 0·04, 0·08), all findings *P* < 0·001 Treatment group increased mean daily frequency of consumption of: fruit (fresh, frozen, canned), (*P* < 0·001, 95 % CI: 0·06, 0·14) vegetables (*P =* 0·048, 95 % CI: 0·00, 0·06), brown rice and cooked wholegrains (*P* < 0·001, 95 % CI: 0·01, 0·02), wholegrain bread and tortillas (*P =* 0·049, 95 % CI: 0·00, 0·07)	–
Chiappone, 2021^(46)^	Frequency of food pantry use (*n* 563) households:^*^ 29 % frequent users 33 % semi-frequent users	Frequency of pantry use significantly associated with food insecurity, (*P* < 0·001) Frequent food pantry users: 45 % severely food insecure 40 % moderately food insecure 6 % marginally food insecure Semi-frequent users: 37 % severely food insecure 40 % moderately food insecure 12 % marginally food insecure	Consumption of foods with added sugar ≥ 1 time per day greater in frequent food pantry users (OR 2·14, 95 % CI: 1·33, 3·44) and semi-frequent users (OR 1·57, 95 % CI: 1·00, 2·46) compared to non-users	–
Fan, 2021^(47)^	Use of charitable food aid during a 7-day survey week	Statistically greater numbers of charitable food aid users were food insecure (48 %) compared to non-users (28 %), *P* = 0·001^†^	People accessing charitable food aid obtained 28 % of their food from food aid Significantly greater proportion of non-starchy vegetables (0·16 [sd: ±0·03] *v*. 0·08 [sd: ±0·02], *P =* 0·018) obtained by people accessing charitable food aid compared to non-users A non-significant increased trend in proportion of meat and beans (0·57 [sd: ±0·11] *v*. 0·34 [sd: ±0·06], *P* = 0·051) obtained by people accessing charitable food aid compared to non-users	–
Loopstra, 2012^(49)^	Use of a food bank in the previous 12 months 23 % of families used a food bank 15 % of families visited a food bank between 1–5 times compared to 8 % visiting between 6–12 times	Odds of using food bank at 12 months follow-up increased with severity: Moderately food insecure (OR 3·21, 95 % CI: 1·26, 8·18) Severely food insecure (OR 3·75, 95 % CI: 1·18, 11·90) Food security status at 12 months: • Food secure 6 % • Marginally food insecure 11 % • Moderately food insecure 25 % • Severely food insecure 39 % Families using a food bank at baseline and follow-up (*n* 54), 41 % were severely food insecure at baseline and remained so at follow-up Families no longer using a food bank at follow-up (*n* 31), only 7 % reported no longer being severely food insecure Families accessing a food bank only at follow-up (*n* 30), 43 % reported being severely food insecure at both baseline and follow-up	–	–
Palakshappa, 2021^(52)^	Follow-up survey between 3–6 months, parents/guardians visited the site on average 0·93 ± 1·38 times in the previous 4 weeks 16 parents/guardians participated in the cooking classes	Qualitative results (3 sub-themes): • Scarce resources: limit foods/meals that could be provided to children • Generational care of children: intergenerational tension of ability to feed every age group in the household • Meals on the weekend: Food availability at weekend limited due to children not having school meals	Non-significant trend in increased children’s average daily fruit and vegetable (including dried beans, French fries/fried potatoes and tomato sauce) intake: Baseline = 3·39 (sd ± 9·02) Follow-up: 3·88 (sd ± 9·44, *P =* 0·41)	No statistical difference in mean parental anxiety scores from baseline (50·0, sd ± 9·85) and follow-up (50·7 sd ± 8·19, *P* = 0·51)
Rizvi, 2021^(50)^	Food bank use of ≥ 3 times in the previous three months: 52 % at baseline 51 % at 6-months 42 % at 12 months 40 % at final 18 months (final follow-up) Baseline 20 % visited a food bank twice and 23 % once in previous 3 months 9 % of participants accessing food banks over the long term accounted for 65 % of all food bank visits	From baseline to18 months follow-up: • Severely food insecure participants decreased by 14 points (39 % - 25 %) • Food-secure participants increased by 7 % points (11 % - 18 %) Aggregated data of all four waves and frequency of food bank visits: ≥ 3 times in the previous three months 47 % severely food insecure 50 % moderately food insecure 45 % marginally food insecure Twice in previous 3 months: 21 % severely food insecure 18 % moderately food insecure 17 % marginally food insecure Once in previous 3 months: 22 % severely food insecure 23 % moderately food insecure 24 % marginally food insecure Significantly improved food insecurity score with: • Food banks located in community resource centres offering additional health and social services: *β* 0·59 (CI: 0·99, 0·19, *P* < 0·01) • Choice-based food bank models: *β* 0·53 (CI: 0·89, 0·17, *P* < 0·01)	–	Mean score (sd): • Baseline 40·2 (11·3) • 6-months 40·4 (11·7) • 12 months 40·8 (13·9) • 18 months 41·6 (11·9) Significant improvement in mental health score between waves 1 and 4 by 1·4 points (*P* < 0·001) Relationship between increasing severity of food insecurity and poorer mental health scores
Wright, 2018^(51)^	All children in the elementary school received a backpack containing food provisions for the weekend every Friday during the academic year	Parental surveys showed a non-significant trend in lower food insecurity at the end of programme (*P =* 0·081) Qualitative survey: 2nd theme identified improved food security leading to greater food availability for the family	–	Parental surveys reported greater child anxiety (*P =* 0·013) and sadness (*P* = 0·010) at the end of the programme Children did not report any negative or positive mental health responses Parental qualitative feedback reported less stress and anxiety associated with not having food. Child reports also stated the backpack helped the family to have more food
Zigmont, 2022^(48)^	Mobile pantry and dinner provided for children during summer school holidays For participants with children: 46 % of respondents planned to use the service: Every weekday 46 % A couple of times or once a week 39 % Less frequently 16 %	Respondents who used the mobile pantry in the previous year: 41 % food insecure 59 % food secure Sufficient food available for food insecure households compared to food secure: Strongly agree 45 % Agree 39 % Disagree 42 % Strongly disagree 50 % Easier to feed the family for food insecure households compared to food secure: Strongly agree 56 % Agree 35 % Disagree 33 % Strongly Disagree 63 %	Helped food insecure families eat a healthier diet compared to food secure: Strongly agree 58 % Agree 36 % Disagree 33 % Strongly Disagree 57 %	–

* Food pantry use categories: semi-frequent user – once or twice a year and some months but not every month; frequent user – once a month, once a week and multiple times a week^(46)^.

† Attributes used to match CFA clients to non-clients included age, sex, marital status, race/ethnicity, education, household size, number of children in the household, number of seniors in the household, whether the household lived in rural areas, monthly household income before tax, whether the household was food insecure.

### Food insecurity

Three studies showed food insecurity prevalence was reduced in households where food aid was utilised^(48,50,51)^. Results from the cluster RCT^(45)^ with 2859 participants show adult food insecurity significantly reduced by 2·8 % points (*P =* 0·002, 95 % CI: –4·8, –0·9) and household food insecurity by 2·4 % points (*P =* 0·003, 95 % CI: –4·1, –0·6) at the first 12-month follow-up. However, no significant difference remained in adult or household food insecurity at the final 18-month follow-up.

The Ottawa cohort^(50)^ included only food bank users (*n* 401). Food bank use of more than three times in the preceding three months decreased over the four waves of data collection: baseline (52 %), 6-month (51 %), 12-month (42 %) and 18-month (40 %). At the end of 18 months, food-secure participants increased from 11 % to 18 %, and severely food insecure decreased from 39 % to 25 %. However, accessing food banks did not appear to be effective as participants with more than three food bank visits remained severely food insecure (47 %), moderately food insecure (50 %) and marginally food insecure (46 %). There were significant reductions in food insecurity by visiting food banks in a community resource centre providing additional health, social and welfare services (*β* 0·59, CI: 0·99, 0·19, *P* < 0·01) and choice-based models in which users choose their food items (*β* 0·53, CI: 0·89, 0·17, *P* < 0·01)^(50)^.

In Loopstra and Tarasuk’s cohort of 371 low-income families in Toronto, only 23 % of families used a food bank^(49)^. Odds of using a food bank at the 12-month follow-up increased with severity of food insecurity; moderately food insecure (OR 3·21, 95 % CI: 1·26, 8·18) and severely food insecure (OR 3·75, 95 % CI: 1·18, 11·90). Among participants using a food bank at baseline and follow-up (*n* 54), 41 % were severely food insecure and remained so at follow-up, with only 13 % no longer reporting severe food insecurity. Of those who no longer used a food bank at follow-up (*n* 31), only 7 % reported no longer being severely food insecure and 13 % reported being newly food insecure.

Evaluation of a backpack programme at a public school in Florida (*n* 120 students, 52 parents)^(51)^ showed a small but non-significant trend in improved parental food insecurity reduced from 2·63 ± 0·166 at the beginning of the school year to 1·81 ± 0·180 at the end of the school year, *P =* 0·081). Qualitative feedback supports the finding as parents stated more food was available for the family.

Cross-sectional survey responses from 153 individuals participating in a summer mobile pantry and supper programme in New Haven, USA^(48)^ demonstrated positive results. Sixty-eight per cent of participants attended with children, of whom 65 % reported it is generally more difficult to feed their family during the summer holidays when children do not receive school meals. The programme proved modestly effective as 37 % of participants agreed it was easier to feed their family compared to 26 % who disagreed. Forty-five per cent agreed they could obtain sufficient food from the programme. However, 13 % of food-insecure participants agreed the programme makes it easier to feed their family compared to 24 % who were food secure. A smaller proportion of food-insecure participants (17 %) reported obtaining enough food compared to 27 % of food-secure respondents.

### Diet quality

Diet quality was better for households using some form of food aid^(44,47,52)^. For instance, children receiving the food parcel delivery in the RCT^(44)^ significantly increased daily fruit and vegetable consumption, 0·1-cup equivalents compared to the control group (*P* < 0·001, 95 % CI: 0·06, 0·13) and 0·06-ounce equivalent increase in wholegrains (*P* < 0·001, 95 % CI: 0·04, 0·08). Additionally, frequency of mean daily consumption significantly increased for fruits (fresh, frozen, canned) (*P* < 0·001, 95 % CI: 0·06, 0·14), vegetables (*P* = 0·048, 95 % CI: 0·00, 0·06), brown rice and cooked wholegrains (*P* < 0·001, 95 % CI: 0·01, 0·02). This represented a 5 % increase in fruit and vegetable and 9 % increase in whole grain consumption for households receiving the food parcel.

The weekend mobile pantry and lunch programme^(52)^ resulted in a non-significant increase in fruit and vegetable intake. Baseline daily serving of total fruit and vegetables (including dried beans and tomato and vegetable soup) was 3·39 (sd ± 9·02) and at follow-up was 3·88 (sd ± 9·44), *P =* 0·41.

Charitable food assistance clients obtained significantly more non-starchy vegetables (0·16 [sd: ±0·03] *v*. 0·08 [sd: ±0·02], *P =* 0·018) than non-clients^(47)^. A non-significant increase in obtaining meat and beans (0·57 [sd: ±0·11] *v*. 0·34 [sd: ±0·06], *P* = 0·051) was also observed between clients and non-clients. Clients obtained 28 % of their food from charitable food aid which suggests that food aid utilisation is likely responsible for providing the additional vegetables, meat and beans.

One cross-sectional^(46)^ study of 563 food pantry users in Nebraska observed a negative impact of pantry access on diet quality. Greater odds of consuming foods with added sugar ≥ 1 per day were reported in frequent (OR 2·14, 95 % CI: 1·33, 3·44) and semi-frequent (OR 1·57, 95 % CI: 1·00, 2·46) food pantry users compared to non-users^(46)^. However, this represents food items obtained from all sources, not only the food pantry, indicating participants’ overall dietary intake. In the mobile pantry with supper programme^(48)^, participants were asked if the programme helped them eat healthier, with 43 % agreeing. However, only 15 % of food-insecure respondents agree the programme helps them eat healthier, compared to 27 % of food-secure participants. Dietary intake data was not collected; therefore, it cannot be deduced which foods improved diet quality or establish any statistically significant improvements.

### Mental health

Three cohort studies reported mental health outcomes^(50–52)^. A small increase in mean perceived mental health scores measured using the 12-item Short-Form Health Survey from 40·2 ± 11·3 at baseline to 41·6 ± 11·9 at the end of the 18-month study period (*P* < 0·001) was reported in the Ottawa cohort^(50)^, demonstrating an improvement. The scores are measured on a continuous scale from 0 to 100, with higher scores indicating better perceived mental health. Lower mean mental health scores were observed with greater severity of food insecurity. Participants who were marginally food insecure scored 44·5 ± 12·2, moderately food insecure 39·6 ± 11·4 and severely food insecure 35·8 ± 10·8. The mobile pantry and weekend lunch programme^(52)^ reported no change in parental mean anxiety scores from baseline (50·0 ± 9·85) to follow-up (50·7 ± 8·19, *P* = 0·51). A score of 50 in the Patient-Reported Outcomes Measurement Information System 8-item Anxiety Short-Form reflects a mean anxiety score for the general population and indicates no depression. Survey responses from parents in the backpack programme at a public school in Florida (*n* 120 students, 52 parents)^(51)^ reported greater child anxiety and sadness at the end of the programme but the children did not report any sadness or anger.

Many programmes reported that parents expressed relief^(48,52)^ from financial pressure and obtained more fruit and vegetables. Children reported being grateful, enjoying healthier foods and trying new foods^(44,45,48,51)^. Parents were appreciative of the healthier food items^(44,48,52)^, convenience^(44,45,48)^ and relief knowing food aid was available locally^(48,51,52)^. However, people did not take full advantage of the food aid. In the RCT^(44)^, only 65 % ordered a parcel in one of the intervention months. The mobile pantry and children’s lunch lost 50 % of their sample due to attrition^(52)^. Sixteen per cent of participants in the other mobile pantry programme stated they would visit the pantry less than once a week^(48)^. The longitudinal analysis^(50)^ also lost 67 % of their baseline sample who accessed food banks. It is unclear why some participants did not fully engage with the programmes or access food banks even though positive feedback was provided.

## Discussion

Food aid use was associated with improved food security and diet quality in some of the included studies. Food bank models offering additional support such as community programmes, health and social services, cooking classes and a free meal for children, client-choice-based models and programmes providing convenient access were more likely to be associated with improved food security and diet quality. Parents also reported that feeding their families with sufficient and healthy foods was easier after accessing food aid.

The findings from this review show that greater severity and persistent food insecurity^(46,47,49,50)^ were often experienced by more frequent food aid users. Likely, a proportion of people accessing food aid in the cross-sectional studies were experiencing food insecurity when surveyed, hence the requirement for food aid assistance. This is a limitation of the included cross-sectional studies, and with this risk of possible reverse causality, the results must be interpreted cautiously.

A qualitative follow-up 6 months after the original study completion of 11 participants found that 10 continued to regularly rely on food banks and stated quality, choice and insufficient quantities of food remained a problem^(58)^. This aligns with research showing that food banks minimally alleviate food insecurity^(59)^ with many people relying on them long-term^(60,61)^. Food banks were not intended to be a long-term intervention; however, they are becoming entrenched in the food environment^(62)^.

Established barriers to accessing food banks include physical access, distance and lack of transport, short opening hours and long queues^(49,63)^. Additional obstacles include not meeting personal food preferences, cultural or religious requirements, receiving insufficient or poor-quality food^(49,58,64–66)^. Qualitative research consistently highlights feelings of shame, embarrassment, powerlessness and stigma which negatively impact the mental health of individuals and their families^(13,14,67,68)^. In response to these challenges, some traditional food bank models have evolved to mitigate the associated mental health impacts. Food bank clients describe the choice of food items as a priority^(60)^, and interventions offering choice give greater autonomy to clients leading to improved self-esteem, a sense of control and dignity^(64)^. Such positive mental health outcomes have been reported in the Ottawa cohort^(50)^ in this review, and improved self-sufficiency and reductions in food insecurity are supported in other studies investigating choice-base models and targeted referral services^(69,70)^.

Parents have been shown to shield children from food insecurity by reducing their food intake to provide food for their children, thereby mitigating negative mental health impacts for their children^(17,26,32)^. In turn, parents experience emotional distress that can be detrimental to their mental health^(71)^. Only one study in this review surveyed both children and adults pre- and post-intervention^(51)^. Parental anxiety had a small improvement, but children did not report any improvement in their mental health. This may suggest that overcoming barriers such as physical access, distance, transport and no queuing to receive food aid may also be an effective way to reach households with children and improve mental health.

Results for diet quality were inconsistent. Studies have repeatedly observed diet quality to be low in food bank users^(72,73)^, with low intakes of fruit and vegetables, dairy^(26,74)^ and increased intake of added sugar^(75,76)^. Only one study in this review observed more frequent food pantry use and increased consumption of foods with added sugar^(46)^. Research shows that food parcels are often inadequate with insufficient quantities of nutrient-dense food^(12,77,78)^, likely due to reliance on donations. Food insecurity is independently associated with a poor-quality diet and poor health^(21,26,79)^. Food aid clients disproportionally face difficulties achieving a healthy diet and are at increased risk of chronic disease^(22)^.

One effective intervention identified in this review was the food parcel delivery^(44)^. A more recent study investigating bi-weekly fresh fruit and vegetable home delivery with virtual nutrition education in the USA^(80)^ did not report significant improvements in food insecurity or fruit and vegetable intake. Both studies included recipe cards and nutritional education as additional resources for participants. The difference in the effectiveness could be that the intervention in this review provided five parcels to select from, potentially giving clients a sense of dignity and improved self-esteem^(66)^. Notably, children liked the novelty of receiving a parcel which some referred to as a present and were more willing to try new foods. A systematic review investigating food pantry interventions in the USA corroborates that choice-based models and nutrition education were the most effective at improving food insecurity and diet quality^(34)^.

One study^(52)^ included in this review provided optional cooking skills classes at a local church or community centre which participants enjoyed and stated they learnt new skills. However, many did not use the classes due to schedules or family commitments. This suggests that educational material can be effective; however, the delivery should be either at home, that is online or at the point of food parcel collection for convenience.

An alternative and convenient method to collecting parcels is giving children a backpack with food items during school hours. Although this review found no favourable outcomes, another study reported children had more energy, improved academic performance, school grades and shared food with other family members^(81)^. Reliable and robust studies investigating the impact of such backpack programmes are still needed as the effectiveness of food insecurity and diet quality is mixed and limited^(82–84)^. Although all children in the school received the backpack, another review observed some children feel ashamed or stigmatised at receiving backpacks^(83)^. Not only could this approach lead to negative mental health impacts for children, it can also diminish the effectiveness in settings where a smaller proportion of the school population is eligible. It could be an effective targeted option in schools or areas where most children are eligible for a backpack.

Accessing food aid may temporarily alleviate or reduce the severity of food insecurity. However, other factors such as employment and income likely have a more substantial impact on reducing food insecurity^(16,17)^. Improving employment and income would be a more effective long-term strategy to reduce the need for long-term reliance on food aid^(85)^.

Studies on households with children, including parent’s and children’s individual perspectives, are limited. Therefore, outcomes in children and adults should be evaluated to develop more effective and targeted interventions to benefit the whole household. Due to the differing political and welfare systems in different countries, the limited evidence from the UK and Europe warrants further research to gauge the effectiveness of current interventions in these geographic and diverse socio-demographic populations.

### Strengths and limitations

This review is the first to systematically review quantitative outcomes of how food aid interventions impact households with children. The screening process, quality assessment and data extraction included a second independent reviewer. A comprehensive search strategy was conducted using a wide range of terms describing food aid from the literature enabling relevant studies to be identified.

Limitations of this review include only studies published in English. Therefore, effective or novel interventions published in other languages could not be assessed. Generalisability of the results is limited due to the heterogeneity of the populations, variability of interventions and outcome measures. The majority of studies did not include a comparator or control group. Consequently, it cannot be inferred the outcomes improved as a direct result of the food aid interventions. With the exception of one study, all other included studies were observational designs and thus causality cannot be inferred.

The heterogeneity of reported outcomes did not allow for statistical analysis or a meta-analysis to compare the effectiveness of the interventions. Only two studies were rated as good, suggesting more high-quality studies are needed to provide robust and reliable evidence of the effectiveness of food aid interventions.

### Implications for public health

Food banks rely on donations from the public and surplus food from commercial organisations such as food retailers and restaurants. With the current global rise in the cost of living and inflation, people are less able to donate. Commercial organisations are potentially reducing costs by limiting surplus food leading to fewer donations. Additionally, the economic crisis will likely increase the number of people who require food aid; therefore, immediate action is necessary to support vulnerable households.

The links between poverty, low income and adverse health outcomes, that is the socioeconomic gradient of health, are well researched. The global economic crisis will continue to constrain household budgets. Vulnerable households are at risk of sliding further down the gradient and likely to become food insecure. Consequently, a greater proportion of the population risk consuming a nutritionally inadequate diet leading to a rise in chronic disease. The resultant healthcare costs of managing chronic disease will place additional pressure on health services. Increased poverty and long-term ill health are major public health concerns.

Whilst out of the scope of this review, some of the issues, namely low income, material and social deprivation, and health inequalities require considerably more upstream action. The government must acknowledge the unintended regular and long-term use of food banks, which include less healthy food than households may choose to purchase. Current policies and the welfare system are not meeting the needs of these individuals and families. There is an urgent need to implement changes in the welfare system and to find a way to support charitable food assistance organisations to provide short- to medium-term relief to current and future users or increase welfare benefit payments to increase food security for lower-income households^(86)^.

## Conclusion

Households continue to experience persistent food insecurity. However, models where clients can choose items, food banks in community centres offering additional support and convenient ways to receive food items demonstrated improvements in food insecurity and diet quality. Choice and support should be incorporated into food aid interventions in the absence of increased value of benefits which would support food security.
